# A patient presenting with a perivascular epithelioid cell tumor in the broad ligament: a case report

**DOI:** 10.1186/1752-1947-5-383

**Published:** 2011-08-16

**Authors:** Claire Ross, Sunita Sharma, Onsy Louca, Michelle Scurr, Andrew Hayes, Ian Judson

**Affiliations:** 1 St Mary's Hospital, Imperial Healthcare NHS Trust, Praed Street, London W2 1UL, UK; 2 Barking, Haveridge and Redbridge University Hospitals NHS Trust, Rom Valley Way, Romford, Essex, RM7 0AG, UK; 3 Northwick Park Hospital, Northwest London Hospital NHS Trust, Watford Road, Harrow, HA1 3UJ, UK; 4 Royal Marsden Hospital, Fulham Road, London SW3 6JJ, UK

## Abstract

**Introduction:**

Perivascular epithelioid cell tumors are a family of rare mesenchymal tumors composed of histologically and immunohistochemically distinctive perivascular epithelioid cells. They can originate in any visceral organ or soft tissue and include a range of lesions such as angiomyolipoma, clear cell 'sugar' tumor of the lung, lymphangioleiomyomatosis and clear cell myomelanocytic tumors of the falciparum ligament/ligament teres. Due to their rarity and varied sites and presentation, management of these tumors remains highly challenging.

**Case Presentation:**

A 46-year-old para 2 Caucasian woman initially presented to the general surgeons at our hospital in North West London with abdominal pain. Laparoscopy revealed a right broad ligament hematoma, which was thought to be iatrogenic in origin, from insertion of the Veress needle at the time of surgery, and was managed conservatively. Upon her re-presentation two months later with severe pain, ultrasound scanning revealed the hematoma had increased in size and she underwent a total abdominal hysterectomy and bilateral salpingo-oophorectomy. Histology results from necrotic tissue from the hematoma led to a diagnosis of perivascular epithelioid cell tumor. She was then referred to a tertiary oncology center, where she underwent several further operations in an attempt to debulk the tumor for symptomatic relief of her pain, with limited success. She is now taking the immunosuppressive drug sirolimus, which has produced a modest reduction in tumor size. She is now 47 months on from initial presentation.

**Conclusions:**

A literature search has revealed only six other case reports of broad ligament perivascular epithelioid cell tumors, with varied presentations and management. The longest duration of follow-up was 21 months. Only five other cases of perivascular epithelioid cell tumor managed with sirolimus have been reported. We therefore feel that this report highlights some of the difficulties in diagnosing perivascular epithelioid cell tumors, and sheds light on management strategies for a very rare gynecological tumor in addition to sharing our experience in the use of sirolimus in its treatment.

## Introduction

Perivascular epithelioid cell tumors (PEComas) are a family of rare mesenchymal tumors composed of histologically and immunohistochemically distinctive perivascular epithelioid cells [1]. They can originate in any visceral organ or soft tissue and have been reported in the lung, pancreas, liver, kidney, bone, uterus, vulva, heart, breast, common bile duct, bladder, skull base, skin and soft tissue of thigh and abdominal wall, and include a range of lesions such as angiomyolipoma, clear cell 'sugar' tumor of the lung, lymphangioleiomyomatosis and clear cell myomelanocytic tumors of the falciparum ligament/ligament teres.

The majority of PEComas arise in women, and have been reported in females ranging from 3 to 97 years of age. We report the case of a patient with a broad ligament PEComa whose history highlights some of the difficulties associated with diagnosing and managing these rare tumors.

## Case presentation

A 46-year-old, para 2 Caucasian woman presented to the general surgeons at our hospital with a four-day history of sudden onset severe lower abdominal pain. She had undergone laparoscopic sterilization 13 years previously and had no medical or social history of note. A laparoscopy was performed because appendicitis was suspected; this revealed a right broad ligament hematoma. This finding was attributed to an iatrogenic injury caused by insertion of the Veress needle during the laparoscopy and, in the absence of peritoneal bleeding, conservative management was initially undertaken. Two months later, she re-presented with acute severe right iliac fossa pain and a repeat ultrasound scan revealed an increase in the size of the broad ligament hematoma.

She was referred to our gynecology department and underwent a laparotomy and a total abdominal hysterectomy and bilateral salpingo-oophorectomy (TAH BSO) five months later. Intra-operative findings included a normal uterus and ovaries with a copious amount of blood in the peritoneal cavity coming from a large right broad ligament hematoma. Exploration of the hematoma released approximately 2 L of old blood and strips of yellow necrotic tissue, with no specific bleeding points identified. Within four weeks, whilst awaiting reconfirmation of the histology results, the patient presented with recurrence of severe lower abdominal pain; a repeat ultrasound scan showed a reaccumulation of hematoma in the right paravaginal space. An angiography failed to reveal a specific bleeding point but a further large volume of necrotic material with blood was removed by ultrasound-guided aspiration. A histological examination of the necrotic tissue revealed malignant polygonal tumoral cells with ample clear or finely granular eosinophilic cytoplasm, focally large pale nuclei with prominent nucleoli and scattered mitoses, with some abnormal forms. Immunohiostochemistry results were positive for HMB45, melan A, caldesmon and smooth muscle actin, leading to a final diagnosis of broad ligament PEComa.

Further management was undertaken in a tertiary oncology center (Royal Marsden Hospital, London), and two months after the TAH BSO, a full body computed tomography (CT) scan revealed a 10 × 10 × 10 cm pelvic mass to the right of the midline with a satellite lesion on the left. In addition, a right hydronephrosis secondary to the mass was diagnosed and managed with nephrostomy and ureteric stent insertion. The patient underwent palliative radiotherapy, after which the nephrostomy was removed. Her pelvic pain did not respond to the radiotherapy and has remained the main issue.

At 10 months after her initial presentation, an examination under anesthesia (EUA) and cystoscopy was performed. Operative findings included a large pelvic tumor closely related to the superior and anterior aspects of the vagina and distorting it. The bladder mucosa appeared normal except for radiation changes. At this stage, further surgery was considered to be a hazardous option and symptomatic management was continued.

Four months after the EUA and cystoscopy the pain had become unbearable for our patient and she underwent an EUA, sigmoidoscopy, cystoscopy and laparoscopy, which revealed a large retroperitoneal tumor on the right pelvic side wall extending down the right vaginal wall and distorting the vagina and bladder. No tumor breach was seen on cystoscopy or sigmoidoscopy. A fistulous connection was found between the tumor and vagina and the bowel was adherent to the tumor mass. A histological examination of the tumor showed features similar to the first sample, with scattered malignant epithelioid malignant cells with enlarged hyperchromatic pleomorphic nuclei containing abundant finely granular eosinophilic cytoplasm and distinct cell boundaries, and was consistent, as before, with malignant PEComa. There were no malignant cells in the peritoneal washings sent.

One month later, repeat CT imaging showed no tumor progression over the previous three months and at this stage her symptoms were tolerable with opioid analgesia. However, four months after this, she developed left-sided hydronephrosis and opted to have a ureteric stent rather than other surgical management options.

The patient agreed to consider the option of sirolimus one month after undergoing stenting of her left ureter, based on a report of clinical benefit in a small series of patients with PEComas treated in the USA [2]. It was hoped that treatment with sirolimus might result in sufficient tumor shrinkage to facilitate a subsequent operation.

Over the subsequent six months, she required numerous hospital admissions with urinary tract infections. During one of these episodes, she was admitted to the coronary care unit with dilated cardiomyopathy and was diagnosed with nephrogenic diabetes insipidus requiring treatment with desmopressin.

The patient remained symptomatic with low abdominal and deep pelvic pain and multi-drug resistant recurrent urinary tract infections, along with radiation induced colitis. A CT scan performed a year after starting sirolimus showed a modest reduction in tumor size and the findings were unchanged on further imaging two months later. Three months after this, she underwent surgery in the hope of resecting the tumor. Unfortunately, extensive fibrosis was found making it impossible to identify the pelvic vessels, hence the tumor resection was abandoned. The fibrosis extended up to the kidneys but the ureters were freed from the fibrous tissue and an ileal conduit was fashioned enabling the ureteric stents to be removed. Since this time there has only been one minor episode of urinary tract infection, but pain continues to be a problem.

**Figure 1:**
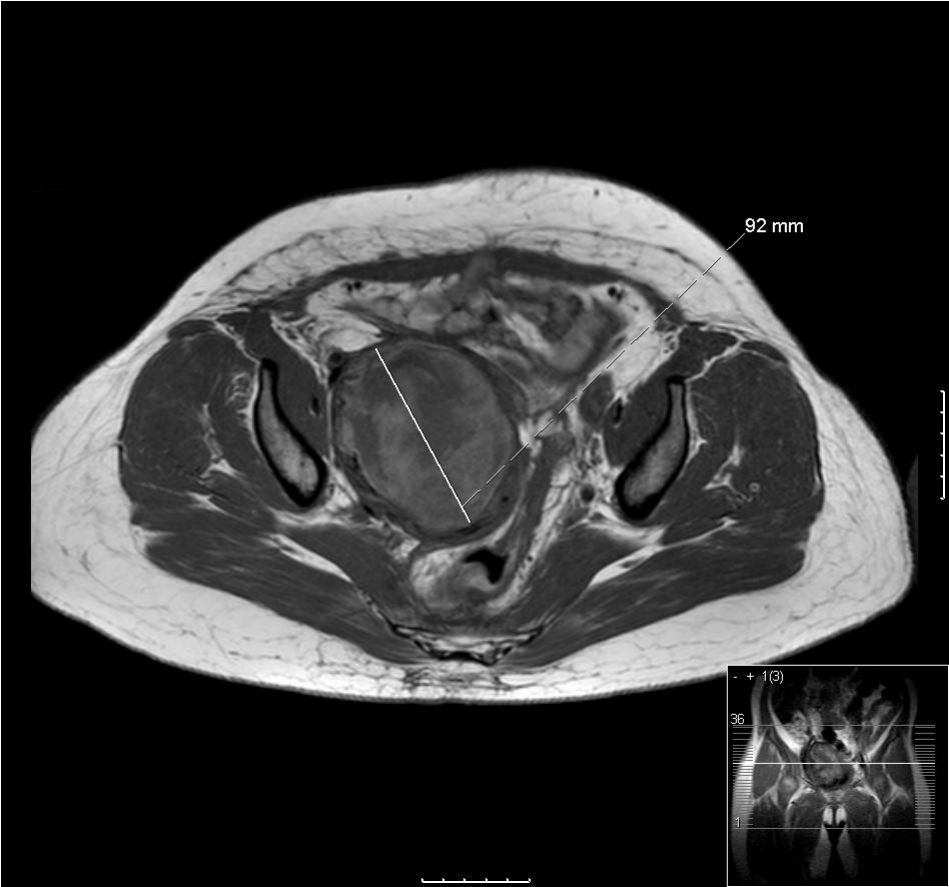
**MRI scan of the pelvis prior to starting sirolimus, following total abdominal hysterectomy and bilateral salpingo-oophorectomy (TAH BSO), demonstrating the perivascular epithelioid cell tumor (PEComa) measuring 92 mm**.

**Figure 2:**
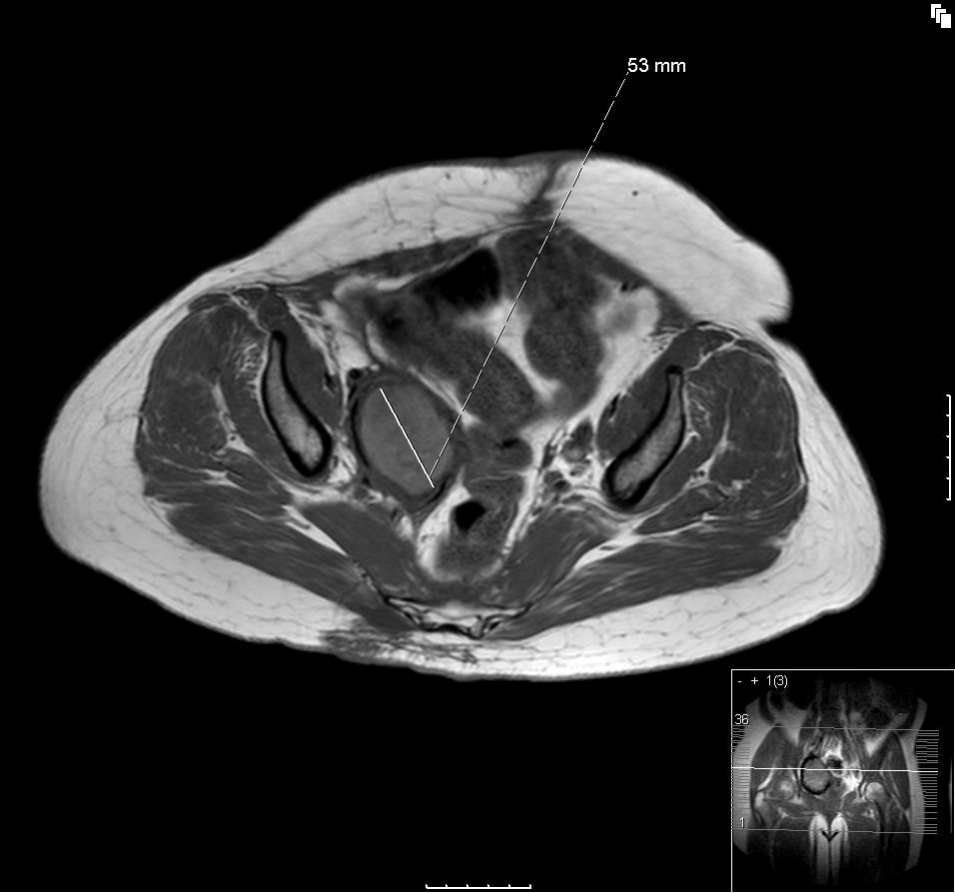
**MRI scan of the pelvis after two years on sirolimus, demonstrating a reduction in size of the perivascular epithelioid cell tumor (PEComa) (measuring 53 mm) following treatment with sirolimus**.

A recent MRI scan (see Figures 1 and 2) shows that over the entire treatment period with sirolimus, a substantial degree of tumor shrinkage has occurred, from a maximum diameter of 92 mm a year after initial presentation (prior to starting sirolimus) to 53 mm following two years of sirolimus treatment. The patient has continued with sirolimus treatment, currently at a dose of 4 mg daily, 5 mg being associated with mouth ulceration.

## Discussion

A diagnosis of PEComa is made on the basis of histological and immunohistochemical findings. Basic characteristic features of perivascular epithelioid cells include abundant clear to eosinophilic granular cytoplasm with an immunoprofile of HMB45 positivity and lack of expression of S-100 protein, though the latter is positive in a minority of cases. Other features include expression of another melanoma-associated antigen melan A, coexpression of muscle cell markers without cytokeratin expression and the presence of melanosomes or pre-melanosomes. In this case the tumor cells expressed HMB45, melan A and smooth muscle actin.

PEComas of the gynecological tract account for approximately 40% of reported cases [3]. In the pelvis, the uterine body appears to be the most prominent site of this rare tumor, with smaller numbers of PEComas reported in the broad ligament, pelvic soft tissue, cervix, vagina and the urinary bladder [4-7].

Morphologically there are two types, epithelioid and spindle cell. In the epithelioid type the cells have clear to lightly eosinophilic, finely granular cytoplasm and may form thick-walled pseudo blood vessels. The spindle cell type, which has fascicles and nests of spindle cells with clear to lightly eosinophilic cytoplasm arrayed around an elaborate capillary sized vascular network, forms the majority of cases [4].

Uterine PEComas are characterized by perivascular epithelioid cells that are HMB45 positive/S-100 protein negative with abundant clear to eosinophilic granular cytoplasm, but there may be an overlap with some epithelioid smooth muscle tumors of the uterus making the initial diagnosis difficult [8]. The patient's lesion is epithelioid in nature. Other differential diagnoses that should be excluded in uterine lesions are endometrial stromal sarcoma and paraganglioma [9].

The patient had a broad ligament PEComa, of which we were able to identify six other reported cases on a literature search of English language articles performed using the search words of 'broad ligament' and 'PEComa' in January 2011 [3,7,9-12]. The age at presentation in these reports ranged from 12 to 51 years. Reported clinical presentation varied from incidental finding of abdominal mass, lower abdominal pain and/or leg pain to abnormal uterine bleeding in a young woman. The tumor typically achieves a large size by the time of diagnosis, representing the difficulty of clinically recognizing these tumors, as was seen in our patient's case.

The current classification of PEComas is based on the morphology and the location of the tumor but this is not an indicator of prognostic outcome. To overcome this, Folpe *et al. *have proposed criteria for classification of these tumors into three groups: benign, of uncertain malignant potential and malignant, based on tumor size, level of infiltration, nuclear grade, cellularity, mitotic rate, necrosis and vascular invasion [3]. Using these criteria, the patient's histology and immunohistochemistry (as detailed above) favor the lesion being classified as a malignant PEComa, and although the tumor has not metastasized, it has behaved in a locally malignant fashion. However, long-term follow-up of a larger number of patients will be required prior to the proposed classification being used as a prognostic indicator.

Duration of follow-up in the literature has varied from eight to 18 months; it is now 47 months since this patient's TAH BSO with persistent evidence of tumor following an initial diagnosis of malignant PEComa.

Reported management mainly includes surgical excision, but this ranged from removal of mass, salpingo-oophorectomy to total abdominal hysterectomy and bilateral salpingo-oophorectomy. In addition, one patient had pelvic radiation along with the TAH BSO and another required re-excision, though local recurrence occurred in three out of six patients [3,11,12]. Our patient underwent initial TAH BSO, followed by USS-guided (ultrasound scan) aspiration of hematoma, pelvic radiation and a trial of sirolimus, which has resulted in some tumor shrinkage that has continued. She has remained symptomatic throughout, mainly from pelvic pain.

Sirolimus is an immunosuppressive agent approved by the US Food and Drug administration for use in the management of renal transplant, renal cell carcinoma and acute myeloid leukemia [13,14]. It inhibits the activation and proliferation of T lymphocytes in response to stimulation by antigens and cytokines (interleukin (IL)-2, IL-4, and IL-15) while also inhibiting antibody production. At a cellular level, it inhibits the activation of the mammalian target of rapamycin (mTOR), a key regulatory kinase, leading to suppression of cytokine-driven T cell proliferation and inhibition of cell cycle from the G1 to the S phase. Sirolimus (rapamycin) is of course the archetypal inhibitor of mTOR, which in cancer cells is associated with increased angiogenesis, stimulation of protein synthesis and inhibition of cell death. PEComas are associated with mutations or deletions of tuberous sclerosis associated genes *TSC1 *or *TSC2*, which act as tumor suppressor genes by regulating mTOR. Their loss results in upregulation of the mTOR pathway. An encouraging recent case series of three patients with advanced malignant PEComa treated with sirolimus reported radiological response and disease stabilization [2]. There was evidence in archive material of changes in *TSC1 *or *TSC2 *in these tumors but these findings were inconsistent and indicate the need for further investigation of the mechanism of mTOR activation in these diseases [2].

Italiano *et al. *also reported a series of two patients with malignant metastatic uterine PEComas, one treated successfully with the mTOR inhibitor temsirolimus (now disease free following resection of her pulmonary metastasis) and the other showing a partial response followed by disease progression, with the temsirolimus being stopped at 22 weeks because of this [15].

Subbiah *et al. *subsequently reported a case of a 58-year-old woman who underwent surgical resection of a malignant PEComa arising from the retroperitoneum above the right kidney. She subsequently developed hepatic metastases whilst on doxorubicin chemotherapy. Following radiotherapy and further chemotherapy, she was treated with temsirolimus but her disease continued to progress and at the time of reporting, she had elected to undergo palliative care [16].

A radiological response to sirolimus was seen in our patient with epithelioid PEComa, as demonstrated by the MRI images shown in Figures 1 and 2.

## Conclusions

In summary, PEComas are rare gynecological tumors, associated with activation of mTOR. Their clinical behavior and the precise molecular mechanisms driving these diseases require further investigation. More thorough reporting and long-term study of all known cases will help us to fully understand, define and manage these tumors. In particular, with regard to mTORC1 inhibitors, accurate histological description of tumor type (epithelioid or spindle cell) would be valuable in further defining and targeting the use of this agent.

## Consent

Written informed consent was obtained from the patient for publication of this case report and any accompanying images. A copy of the written consent is available for review by the Editor-in-Chief of this journal.

## Competing interests

The authors declare that they have no competing interests.

## Authors' contributions

CR and SS wrote the manuscript. SS and OL were involved in the initial management of our patient. IL, MS, AH and IJ were involved in the subsequent management of our patient. All authors contributed to and critically reviewed the final manuscript.
